# Complimentary Letters, and Those That Are Not

**Published:** 1870-07

**Authors:** 


### Our Correspondence.

### COMPLIMENTARY LETTERS, AND THOSE THAT ARE NOT.

#### "Tit for Tat."

AN INTERESTING LETTER FROM SCOTLAND AND GERMANY.

*A Letter from a disgusted individual in Broome Co. He appropriates mail matter not his own, and attemps to annihilate the Editor of the Bistoury—just hear him.*

UNION CENTER, March 26, 1870.

DR. THAD. S. UP DE GRAFF—Sir:

I am a man forty-seven years old; have done business for myself ever since I was fourteen; have for several years and am at present taking seven different Journals, for which I always have and do *invariably* pay in advance, and never received a dun until to-day. My boy brought from the Post Office a dun from you, claiming a years subscription for the BISTOURY, a Journal I had never, to my knowledge seen or heard of before.

Accompaning the dun was two Numbers of the BISTOURY; No. 3 of Vol. 5 and No. 1 of Vol. 6; which I return to you with your dun, and blank. As for Vol. 5 I shall not pay for it, for I never subscribed for, and never received the Journal; and I want you to understand *implicitly*, never to send me any more BISTOURYS until I *subscribe* and *pay* for it.

You profess to expose humbugs, &c., now if this is a game to humbug fifty cents out of all who are green enough to pay it, perhaps you will make a little, but not out of me.

I now know what and where the BISTOURY is, and when I want it, I shall *subscribe* and *pay* for it in advance. You, nor any one else will ever have opportunity to dun me for arearages on subscription. The fifty cents is of no account with me, but I wont be *gulled* if I know it.

WM. TWISS, not TWIST,
UNION CENTRE, Broom Co., N. Y.

— To which we humbly reply as follows:

WM. TWISS—not TWIST, UNION CENTRE, N. Y.

*Indignant Sir:*—It is the deliberate opinion of the Editor of the BISTOURY, that a man who has advanced to the age of 47 years, and who is the constant reader of seven different journals, for all of which he has the good sense to pay for in advance, ought to be sufficiently familiar with the characters representing his own name to know that "T-w-i-s-t" does *not* spell "Twiss!"—notwithstanding you have twisted it into so doing. It occurs to us that you had better subscribe for seven more journals—being careful to pay for the same in advance—so that by the time you are 47 years older you may possibly learn that it isn't a gentlemanly act either to receive mail-matter evidently not intended for you, or to write abusive letters to those who have done you no intentional wrong.

— Here is another chap who has appropriated some gentleman's BISTOURY to his own use,—hear him splurge:—

EDITOR BISTORY—

*Sir*:—I think it is time to inform you that I am not fifty cents (50c) behind in act with you You are *very* kind to send a bill to be filled out and want the am't due you for subscription to the BISTORY for the year Now Mr Editor when I subscribe for your BISTORY I will not wait for a duning letter but as I never did subscribe for any such as you have been sending this way I do not signefy my wilingness to pay you nor to continue any subscription to the BISTORY.

I rather you have been sending it to the wrong person Please send your duning letters to your subscribers and not to me

Respctly
FRANK S. MILLER,
MITCHELL'S CREEK, Tioga Co. Pa.

P. S. I have a brother at Genesse Station N. Y. and perhaps he is the man his name is C Miller

— To this gay and festive youth we reply as follows:

Your orthography is worse than your abuse, while your impudence surpasses both. It is apparent to us that a gushing youth who is competent to write a miserable letter, *should be* possessed of sufficient intelligence to know better than to appropriate mail-matter he *knows* is not intended for him. You confess your name to be Frank S. Miller; by what right do you reply to a circular addressed to C. Miller? The BISTOURY was sent to a gentleman and *not* to you! Snobs never read our journal—we know better than to send it to such. Please don't gush any more, bub,—you will spoil your temper.

— In addition to the above effusions, we have received two other letters, in answer to our circular, one of which is from an ignoramus who calls himself Dr. (?) White, and who deals death and physic to his victims at Trumbull's Corners, N. Y. He also replies to the circular addressed to his brother, a gentleman and physician. We have not space to devote to this beautiful specimen of composition, else we would give it to our readers. The other is from a chuckle-headed Post Master at Reed's Corners, N. Y. He grumbles that we should send him notice to pay for one year's subscription to the BISTOURY, after having had it five years for nothing.

In contrast to the above we select one from very many complimentary letters which we have received from physicians and others, from all parts of the country. We thank our friends for their kind exertions in behalf of the BISTOURY. At the same time we afford our enemies an opportunity to vent their spleen, as above. We hope all parties will feel entirely satisfied with our efforts to please them.

---

KISEVILLE, Kentucky, March 17, 1870.

THAD. S. UP DE GRAFF, M. D., Elmira, N. Y.

*Dear Sir:*—I am still working for the BISTOURY, the best medical journal in the world, of its size.

The condensed form in which it is presented to the public, makes it a most excellent magazine for every household. Let every physician of our high and Noble Alopathic Order, introduce it to his patrons and the community in which he lives; it will sustain him in his great calling, enlighten his friends and save them from the many quacks and humbugs which are daily thrusting themselves upon the most honest and unsuspecting classes of our country.

Go on, and may God bless you and reward you in your good work. I send $1,50 for three more subscribers.

Yours Fraternally,

F. L. N., M. D.

---

— Here follows a jolly sort of a letter, from an old gentleman, who is in possession of a good appetite and an ample digestive apparatus:—

WARREN, R. I., Feb. 28, 1870.

*Sir:*—Enclosed please find the amount stated in your prospectus that will procure the yearly visitation of your valuable Journal. I am free to say that the idea therein stated, with regard to the treatment of Children, and Boys, especially, meet my views fully and completely. Were those directions carried out in full, the world would be soon rid of all dwarfs, both physically and intellectually; their minds and bodies are cramped and dwarfed, before they are half matured, by the common maltreatment they receive.

With regard to Ishmael's familiar treatment of the stomach, of that I cannot speak so fully, not being yet fully convinced as to which head I should come under; whether the Ostrich, Kangaroo, Turkey, or Goose; more probably the goose, having in my possession a Gizzard, that has never yet failed to masticate all, and everything I have ever deposited in it, without fear of pain, or remorse of conscience; therefore I feel my inability to examine and analyze the subject. I never, however, attempted to eat raizors, and doubt my ability to digest them properly; my greatest fear is that I may not obtain good and wholesome food enough to sustain my physical frame while I dwell in the flesh. I must confess it to be a great blessing, that many do not enjoy, of having plenty to eat, a good healthy appetite, with good masticating powers and digestive organs to do the work, free from all pain.

Very Truly Yours, S. M.

---

EDINBOROUGH, Scotland,
April 13, 1870.

MY DEAR DOCTOR:—I sailed from New York Oct. 23rd, and after (with some little exception) a pleasant passage, arrived at Glasgow, Nov. 5th. As regards the voyage I might observe, that we encountered a gale off the Irish Coast that tumbled things fearfully. The cargo was shifted to the starboard side, putting the ship almost on her "beams' end." A "cap" was displaced, uncovering a "coal-bunkes-port," and in came the water and out went three of the fires in a twinkling. Matters began to assume anything but an agreeable aspect. The ship was careened badly, and the sea rolled over her every moment, as if to take her bodily into its fathomless maw. But the cap was finally replaced, and towards night the storm, having exhausted its fury, abated, and the good ship proceeded on her way. I can tell you, Doctor, that a storm at sea is a very sublime affair, indescribably so. As for myself, however, I don't care for any more of that sort of sublimity; I am surfeited.

I came over here last Saturday, and shall sail for Hamburg to-morrow. By Saturday of the present week I hope to be at my post in Brunswick.

Edina has many places of interest to the traveler, but my brief sojourn precludes my visiting but a few of them. Yesterday I went to Holyrood Palace so famous in Scottish history. An abbey was erected here sometime in the 12th century, and the ruins are still to be seen. Within it many of the Scottish Kings were buried. During the latter part of the 15th century (about the year 1490) James the fourth, who fell at Flodden, built a palace—the palace of Holyrood—right adjoining the Abbey. His son James the Fifth made some additions, and to-day the old palace is in a good state of preservation. Many important events are associated with Holyrood; but what invests its venerable apartments with thrilling interest, are those events which connect them with the life of Mary Queen of Scots, beautiful but unfortunate woman whose sad history makes us forget her faults, while it awakens in our bosoms only sympathy for her sufferings and tragic death. Here Mary reposed after her return from the gay land of France; here took place her unfortunate marriage with Darnley; here Riccio, her secretary, was murdered while clinging to her very clothes; here also she married the cruel Bothwell, which was the final blow to her peace and happiness in Scotland; and within these walls, at many a royal entertainment, she enchanted all who beheld her, with her loveliness of person and her graceful and accomplished manners

The first room you enter in the palace is the portrait gallery, where are exhibited portraits of the Scottish Kings. Some of them reigned before the Christian Era, and fought the Romans in the time of Julius Cesar.

We next come to Darnley's rooms where the conspirators met on the evening of Riccio's murder. Ascending a flight of stairs we reach Mary's apartments, the center of interest to the visitor. The first room is "Mary's Audience Chamber." It is, I should judge, some twenty-five feet square. The roof is divided into parallel compartments, adorned with initials and armorial bearings of the Kings. The walls are covered with ancient tapestry, now sadly faded. In the room there is a bed which was used by Charles the First of England, while visiting this palace. On this couch reposed Prince Charles the Pretender in 1745; and after the battle of Culloden, the Duke of Cumberland, his conquor, slept on the same pillow. I dare say this bed was a fine thing in its day, but moth-eaten and faded, it looks rather rusty now. It was in this room that Mary had those fierce altercations with that bold, old Reformer, John Knox. There he was more than once summoned to answer to some furious invective against Mary, her Court and her religion. He met her fearlessly and boldly, charged her with infidelity and idolatry, and neither her beauty nor her tears could soften his heart in her behalf. On one occasion he had preached against her contemplated marriage, when she sent for him, and indignantly asked, "What have ye to do with my marriage, and what are ye in this Commonwealth?"—"A subject born within the same, Madam;" was the stern reply; "and, albeit I be neither Earl, Lord nor Baron within it, yet has God made me, how abject soever I may be in your eyes, a profitable member within the same."

As I stood in that room how vividly were those scenes conjured up by imagination! Passing through the Audience Chamber we came to "Mary's Bedroom." The identical bed on which she slept is still there. In it were chairs, a mirror, and a work-box which were hers. A door opens from the bedroom into a small room in one of the turrets. In this little room a door opens upon a private stairway which leads down to Darnley's apartments, and to the foot of the turret (inside). It was by this stairway that the murderers of the Queen's Secretary gained access to her apartments. The murder took place in this wise: Mary would occasionally serve tea to a few friends in the little room referred to. On the evening of march 9th, 1556, while sitting at one of the little parties, at which Riccio was present, Darnley, her husband, entered the room and affectionately placed his arm around her waist, at the same instant the conspirators rushed up the stairway and filled the room, in the confusion upsetting the table, which fell upon the Queen. Riccio clung to Mary for protection, and throwing himself behind her, begged of her to save him; but he was stabbed over her shoulder, and was finally dragged out through her bedroom, receiving fifty-six wounds on the way. Mary pleaded with strong cries for the life of her favorite, but on learning that he was dead she dried her tears and exclaimed, "I will now study revenge." The subsequent murder of Darnley, and her marriage with his murderer Bothwell, showed that she kept her word.

A melancholy atmosphere pervades these apartments, and a strange interest causes the visitor to linger within them. But I have tarried too long.

The house where John Knox lived and died, where he prepared those furious fulminations against Popery, still stands, an object of veneration to the admirers of the bold Reformer.

The castle was the next place of interest that I visited. It stands on a high crag and looks frowningly down on the city which surrounds it. A part of it was built in the 6th century, to resist the invasions of the Danes. The view from its towers is the finest I have ever had. A room is shown where Mary gave birth to James the Sixth, afterwards James the First of England. The crown of Robert Bruce is exhibited, and many other things of interest to the antiquary.

There is so much to be seen that one might spend a week or more about Edinborough in a very pleasant way. I hope to be able to come over next Summer, and visit Melrose Abby, Stirling, Abbot's ford, and the house of Burns, as well as other parts of Scotia. But I must stop here.

If you have patience to read this, Doctor, you will have more of that virtue than I possess. I have written very hurriedly by a miserable light. My excuse for writing so much is, that I know you to be a lover of antiquities. How I wish you were here with me to enjoy these scenes of historic interest.

After our correspondent's return to BRUNSWICK he continued his letter as follows:—

"I fear my long silence has led you to regard me as no longer in the flesh, but I hasten to assure you that I am still *in*, and take with most remarkable regularity, my rations of beer, sausages, and sourkrout; and the size of my girth is steadily on the increase. At present I am quite well in body and mind, and find myself at peace with the world and my conscience. Some weeks ago, however, I had trouble. I awoke one fine morning, and found myself unable to get out of bed. My servant thought I had the cholera, and I knew I had *something*, not altogether agreeable to have. After consultation of the household, it was determined, with but one dissenting voice, which was my own, to send for a doctor. One was sent for, and soon made his appearance on the ground. He looked at me and smiled, and I looked at him and shuddered. He proceeded to make an examination of the case—felt my pulse, pressed my bowels, and looked professionally down my throat. At the expiration of three quarters of an hour he stepped back, stroked his whiskers, surveyed, sadly, my aching body, from head to foot, and ejeculated "*Schlect*" (bad).

Well, I was under his treatment one week, and lived through it! On my recovery, I told my man that if he ever brought another doctor into my room, I would shoot him, (the man) on the spot. He said, "Ich will nimmer, Herr Consul," and I guess he wo'nt.

Don't think my dear Doctor, that I have an unfortunate prejudice against your profession. I have nothing of the kind. Doctors must live—it's a pity that their patients can't—and I am willing to take my share of suffering at their hands for their support; but I don't want to die in *Brunswick*, and for this reason. It is a deal of trouble and expense to get decently buried in this city, as you may see.—In the first place, each of the bearers are entitled to a bottle of wine and a loaf cake, before he will leave the house with the corpse. In the second place, I don't like the coffins they use here. They are huge black boxes, and when placed on the burial car, they look like the car of Juggernaut, or an ark on wheels. Then again, one has got to make a big dinner for the mourners and invited guests, who are always sure to be numerous, and who require much wine and many cigars. Again, your grave lease expires in twenty years, after which time, if there is anything left of your bones, it will be put away in some unknown corner, and your old resting place will be occupied by a new tenant. For these and other reasons I would prefer not to depart this life in B. * * * *.

Some time since I read an article in an English paper on the subject of execution by beheading. The writer claimed that the head retained consciousness after decapitation. Well, I have a bit of evidence bearing on the question. Last October a man was executed in this city. There were several physicians and other prominent gentlemen present. Immediately after the head had been struck off, one of the gentlemen raised it, and with a large pin stabbed at the eyes, they being open. *An effort to wink was seen by all present, and this could be perceived four minutes after the decapitation.* These facts I have from individuals who witnessed the experiment and who are persons of credibility. Is it not passing strange?

A case of transfusion of blood was reported in our German papers a few weeks ago. I read it in a Brunswick paper. It appears that a man had been suffocated by gas and seemed to be dead. The physicians in attendance tried a number of experiments in their attempts to resuscitate the man but they failed. At last, one of them suggested the transfusion of blood and it was tried. One of the waiters was brought in and tapped, and the warm current was turned into the veins of the dead man. In a short time circulation was restored, the heart began to beat and the man was brought to life, or to be more correct, life was brought to him. As our newspapers are not given to the publication of *canards*, I see no reason why this report may not be regarded as true.

The Parliament of the North German Union has voted to abolish capital punishment, thereby setting an example which I trust will be followed in the United States. There is one thing more that the Parliament should abolish, and that is their large standing army; but they wo'nt do this, because it is the fashion just now in Europe, to measure the importance of a State by the number of armed men it can bring into the field, and influence in the councils of Nations implies a large standing army.

Duke William is well, and to all appearances quite contented with his lot. His *quasi* dutchess is also comfortable. I think I have informed you that his Highness is not married, "according to the statute in such case made and provided;" he is, however, married according to the custom of the dukes. His birthday, which occurs anually, will be celebrated the 25th of the present month. I shall be there. His age will be 64.

Very Truly Your Friend,

D. W. C. S.

The above letter was not intended for publication; but we have taken the liberty of making extracts from it, believing that it will be of interest to very many of our readers. Our correspondent is United States Consul to Brunswick, and is a gentleman of rare culture and attainments. We hope to be able to present a letter to our readers, written purposely for the BISTOURY, by our next issue.

---

Advices received by the Cape mail, in England, state "it is the general opinion in the colony that Dr. Livingston is dead."

---

The proverb, *In vino veritas*, meant something once, but its application now-a-days depends entirely upon your wine merchant.

